# Cereblon调节T细胞逆转PD-1抗体治疗肺癌耐药的研究进展

**DOI:** 10.3779/j.issn.1009-3419.2020.102.49

**Published:** 2021-01-20

**Authors:** 晶晶 郭, 迪 牟, 颖 韩

**Affiliations:** 1 300060 天津，天津医科大学肿瘤医院，国家肿瘤临床医学研究中心，天津市肿瘤防治重点实验室，天津市恶性肿瘤临床医学研究中心，天津市肿瘤免疫与生物治疗重点实验室 National Clinical Research Center for Cancer, Key Laboratory of Cancer Prevention and Therapy, Tianjin's Clinical Research Center for Cancer, Key Laboratory of Cancer Immunology and Biotherapy, Tianjin Medicial University Cancer Institute and Hospital, Tianjin 300060, China; 2 300060 天津，天津医科大学肿瘤医院，国家肿瘤临床医学研究中心，天津市肿瘤防治重点实验室，天津市恶性肿瘤临床医学研究中心，生物治疗科 National Clinical Research Center for Cancer, Key Laboratory of Cancer Prevention and Therapy, Tianjin's Clinical Research Center for Cancer, Department of Biotherapy, Tianjin Medicial University Cancer Institute and Hospital, Tianjin 300060, China

**Keywords:** Cereblon, T细胞, 程序性死亡受体-1抗体, 肺肿瘤, Cereblon, T cell, Programmed cell death receptor 1 antibody, Lung neoplasms

## Abstract

程序性细胞死亡受体1（programmed cell death receptor 1, PD-1）是一种跨膜蛋白，主要表达于T细胞，并与靶细胞上的PD-1配体，即细胞程序性死亡配体1（programmed cell death ligand 1, PD-L1）结合。PD-1作为一种免疫抑制分子，当PD-1与肿瘤细胞上的配体PD-L1结合时，抑制了T细胞的免疫功能，从而发生肿瘤的免疫逃逸，如外周效应T细胞的耗竭导致效应T细胞向调节性T细胞（regulatory T cells, Tregs）转化。为解决这一问题，利用PD-1抗体与T细胞上的PD-1结合，从而抑制T细胞表面的PD-1与肿瘤细胞表面的PD-L1相互作用，进而恢复T细胞杀伤肿瘤细胞的功能。以Nivolumab和Pembrolizumab为代表的PD-1抗体现已被批准用于晚期非小细胞肺癌的一线治疗。但在部分患者中出现了由于肿瘤细胞、T细胞及细胞因子的相互作用导致的耐药，降低了免疫治疗的疗效。因此，如何克服患者的耐药成为了当前待解决的主要问题。研究发现，Cereblon（CRBN）作为DDB1泛素环E3泛素连接酶复合物的底物受体及免疫调节药物唯一已知的结合受体，与CRBN调节剂（cereblon modulatory agents, CMs）结合可以通过上调T细胞的增殖、激活和代谢，发挥T细胞的免疫功能从而逆转PD-1抗体耐药。本文就T细胞的下调导致PD-1抗体治疗肺癌耐药的机制、CRBN调节T细胞的机制及CRBN调节剂治疗肺癌的研究进展进行综述。

## Abstract (en)

Programmed cell death receptor 1 (PD-1) is a membrance-spanning protein mostly expressed in the T cell, and combines with programmed cell death ligand 1 (PD-L1) in the targeting cell. When binding to the ligand on tumor cells, PD-1 as an immunosuppressive molecule, can inhibit the immune function of T cells, thus tumor immune escape. For example, depletion of peripheral effector T cell and accelerate the transformation of effector T cells into regulator T cells. To solve this problem, PD-1 antibody is used to bind to PD-1 on T cells to inhibit the interaction between PD-1 on the T cells and PD-L1 on the tumor cells so that it can restore the function of T cells to kill tumor cell. PD-1 antibodies, such as Nivolumab and Pembrolizumb, are approved as a first-line treatment for advanced non-small cell lung cell cancer. However, due to the interaction of tumor cells, T cells and cytokines, some patients developed drug resistance which reduces the efficacy of immunotherapy. Hence, how to overcome resistance has become a urgent problem. Cereblon (CRBN), a substrate receptor of the DDB1-cullin-RING E3 ubiquitin ligase complex and the only known molecular receptor of immunoregulatory drugs, has been found to reverse PD-1 antibody resistance by binding to CRBN regulatory agents (CMS), exert T cell immune function by regulating proliferation, activation and metabolism of T cell. In this paper, the mechanism of down-regulation of T cells leading to resistance of PD-1 antibody in lung cancer, the mechanism of CRBN regulating T cells, and research progress of CRBN regulator in the treatment of lung cancer were reviewed.

肺癌是全球癌症死亡的主要原因，非小细胞肺癌（non-small cell lung cancer, NSCLC）占肺癌的85%^[1]^。临床上，多数NSCLC诊断时已为晚期。但是，随着免疫治疗的进展，多种免疫检查点[如程序性细胞死亡受体1（programmed cell death receptor 1, PD-1）、细胞毒性T淋巴细胞相关蛋白4（cytotoxic T-lymphocyte antigen 4, CTLA-4）]抑制剂通过增强患者自身免疫系统的功能，直接或间接地抑制肿瘤的发生发展，在肿瘤治疗过程中发挥关键作用，尤其是PD-1/细胞程序性死亡配体1（programmed cell death ligand 1, PD-L1）抗体目前被认为是NSCLC治疗的支柱。Nivolumab是免疫检查点抑制剂（immune checkpoint inhibitors, ICIs）中第一个被批准用于治疗肺癌的药物，Pembrolizumab作为获得美国食品药品监督管理局（Food and Drug Administration, FDA）批准的第一批PD-1抑制剂，二者均显著提高了晚期NSCLC患者的无进展生存期（progression-free survival, PFS）和总生存期（overall survival, OS），同时改善了患者的生活质量。尽管在免疫检查点抑制剂疗法中观察到持久的缓解率，但大多数患者未能从治疗中获益，肿瘤细胞、免疫细胞和肿瘤微环境之间不断的相互作用，使得肿瘤细胞通过免疫消除、相持和逃逸三个连续阶段而逃避免疫监视，发生原发性耐药、适应性耐药和获得性耐药^[2, 3]^。T细胞是介导免疫耐受的主要细胞，增强T细胞的功能可以更好地抑制肿瘤的生长和增殖。肿瘤免疫治疗激活肿瘤内的细胞毒性T淋巴细胞（cytotoxic lymphocyte, CTL）的活性，协助激活淋巴器官中的肿瘤特异性CTL，建立持久有效的抗肿瘤免疫^[4]^。通过对肿瘤患者T细胞的克隆扩增，可以改善免疫检查点抑制剂的临床效果^[5, 6]^。相反，肿瘤特异性T细胞的功能障碍导致肿瘤发生^[7]^，肿瘤浸润性T细胞数量下降的患者预后较差^[8]^。因此，如何重新激活T细胞的免疫功能、逆转PD-1/PD-L1抗体的耐药、提高免疫治疗的疗效仍是当今治疗的难点和热点。最新研究^[9]^发现，Cereblon与CMs（如沙利度胺和CC-885）结合通过调节T细胞的生长、增殖与代谢，上调T细胞的免疫功能，从而逆转PD-1抗体的耐药。本文就T细胞的下调导致PD-1抗体治疗肺癌耐药的机制、CRBN调节T细胞的机制及CRBN调节剂治疗肺癌的研究进展进行论述。

## 1 T细胞的下调导致PD-1抗体治疗肺癌耐药的机制

### 1.1 细胞产物下调了T细胞的生长增殖与活化

在肿瘤微环境中，肿瘤浸润性T细胞会因慢性抗原刺激和炎性肿瘤微环境而衰竭。此外，肿瘤细胞和基质细胞释放多种细胞因子，如吲哚胺2, 3-双加氧酶（indoleamine 2, 3-dioxygenases, IDOs）、外泌体和白介素6（interleukin 6, IL-6）等抑制效应T细胞功能^[10]^。

IDOs是参与色氨酸分解代谢的限速酶，在肿瘤微环境中具有免疫抑制功能^[11, 12]^。IDO将色氨酸氧化为犬尿氨酸，而色氨酸是T细胞活化的必需氨基酸，犬尿氨酸则会抑制T细胞的活化。该反应形成了一个以效应T淋巴细胞和自然杀伤细胞减少为特征的高敏微环境，并导致Tregs和髓源抑制性细胞（myeloid-derived suppressor cells, MDSCs）数量增加^[11, 13]^。此外，肿瘤细胞分泌的IL-6可以激活MDSCs，进而使IDO发挥作用，进一步抑制T细胞的活化^[14-16]^。

肿瘤细胞分泌的外泌体也有类似的功能。外泌体通常为球形，由薄膜包裹，大小为30 nm-100 nm，表面携带有生物活性的PD-L1，可以抑制免疫应答^[17]^。为了研究外泌体PD-L1的生物学功能，将PD-L1转到低表达或无表达的细胞中，发现低表达外泌体PD-L1显著抑制CD3/CD28-诱导的ERK的磷酸化和核因子κB激活T细胞^[18, 19]^。Tregs外泌体miRNA通过抑制环氧合酶-2介导的干扰素4（interferon 4, IFN‐4）通路，强烈抑制Th1细胞活性^[20, 21]^。此外，外泌体PD-L1还可以通过抑制T细胞颗粒酶B的分泌，促进肿瘤的生长^[18]^。肿瘤干细胞上的CD80通过与T细胞表面的CTLA-4结合，不仅抑制了T细胞的活性还降低了T细胞的增殖^[22, 23]^。

### 1.2 细胞因子调节T细胞的功能

肿瘤微环境中产生的促炎细胞因子可以消除抗肿瘤免疫，提高肿瘤细胞存活率^[24]^。肿瘤坏死因子*α*（tumor necrosis factor *α*, TNF-*α*）不仅是促进肿瘤的生长、生存、扩散及血管生成^[25-27]^的重要因子，也是PD-L1触发肿瘤细胞免疫抑制的主要因素。由核因子κB p65诱导的COP9信号转导体5（COP9 signalosome 5, CSN5）对TNF-*α*介导PD-L1稳定癌细胞及抑制PD-L1的泛素化和降解，极其重要^[24, 28]^。PD-L1抑制IFN-*γ*的分泌，并通过减少细胞因子、诱导CD8^+^ T细胞的凋亡和限制T细胞进入非淋巴组织而损害免疫功能^[10, 29, 30]^。肿瘤浸润T细胞产生的促炎细胞因子*IFN-γ*和*JAK*-3突变可进一步驱动PD-L1表达，促进适应性抵抗和免疫抑制^[2, 10]^。

IL-6是一种强效炎症细胞因子，调节T细胞和B细胞的分化和激活，在多种细胞中表现出功能多效性。在某些类型的肿瘤中，IL-6通过IL-6/JAK/STAT3信号通路向肿瘤细胞发送信号，并重塑局部微环境来支持原发肿瘤的生长、生存和侵袭。STAT3在肿瘤浸润性免疫细胞中过度激活，不仅对中性粒细胞、自然杀伤细胞、效应T细胞和树突状细胞产生负调控作用，还可以正向调节Tregs和MDSCs抑制抗肿瘤免疫反应^[31-34]^。在肺腺癌和胃癌患者中观察到高IL-6的表达与患者生存情况呈负相关^[35]^。IL-10可抑制T细胞功能，促进T细胞向Tregs的转化，从而减弱T细胞的抗肿瘤免疫反应^[36]^。IL-22在肺癌细胞与免疫环境相互作用中起调节作用。通过酶联免疫吸附法分析了195例支气管镜灌洗标本IL-22的含量，发现在肺癌患者中IL-22的浓度较高^[37]^。

转化生长因子-β（transforming growth factor-β, TGF-β）是免疫稳态和免疫耐受的重要因子，抑制免疫系统许多成分的功能。TGF-β信号不仅是炎症性疾病的基础，还促进肿瘤的发生。在肿瘤微环境中，TGF-β是免疫抑制的核心，通过破坏DCs功能和招募MDSC、Tregs等免疫抑制细胞，抑制淋巴结中肿瘤抗原特异性CD8^+^ T细胞的诱导及浸润^[38-40]^。TGF-β信号不仅能够导致和维持T细胞耐受，还可以抑制外周CD8^+^ T细胞在外源性刺激下的扩增和活性^[39-41]^。效应Treg细胞表达大量的整合素αvβ8，激活潜伏期TGF-β和肿瘤源性TGF-β。反过来，TGF-β又能够诱导FoxP3表达及Treg细胞的生成^[42]^。

此外，肿瘤细胞通过抗原调节和免疫检查点分子相互作用而抑制免疫作用，抗肿瘤T细胞生成不足、肿瘤特异性T细胞功能不足及T细胞记忆形成受损等机制均可以导致肿瘤免疫逃避^[43-45]^，且内源性T细胞需要通过抗原刺激及细胞因子介导识别和杀死肿瘤细胞。可见，克服T细胞的下调对免疫检查点抑制剂的治疗极其重要^[46]^。

## 2 CRBN调节T细胞的机制

CRBN是一种结合蛋白，广泛表达于前列腺、肝脏、胰腺、胎盘、肾脏、肺、外周血白细胞和大脑的细胞质、细胞核和外周膜中^[47-51]^。人类CRBN基因位于3号染色体3p26.2处，由1, 329个碱基对组成，其中包含11个预测外显子，其中外显子5-7和10-11分别是DNA损伤结合蛋白1（DNA damage-binding protein 1, DDB1）和沙利度胺类免疫调节药物的结合位点^[52-55]^。*CRBN*最初被认为是常染色体中导致隐性轻度智力障碍基因^[53]^，且与DDB1相互作用致畸。有研究^[56]^发现，CRBN在调节肿瘤细胞增殖、新生血管形成、T细胞和NK细胞免疫活性、细胞间黏附分子表达中发挥重要作用。

### 2.1 CRBN调节CD8^+^ T细胞的增殖和激活

CRBN缺乏的情况下，T细胞受体（T cell receptor, TCR）信号相关的基因受到了影响，因此CRBN表达的差异可以调节T细胞对TCR刺激的敏感性^[51]^。在同基因B16黑色素瘤移植研究中，Crbn^-^/^-^小鼠比Crbn^+^/^+^小鼠的肿瘤进展和肿瘤负荷明显减少，且增加了CD44^+^黑素瘤抗原酪氨酸相关蛋白（tyrosinase-related protein, Trp-2）反应性CD8^+^肿瘤浸润性淋巴细胞数量，表现出更好的抗肿瘤活性。通过定量RT-PCR和免疫印迹分析表明，CRBN蛋白和mRNA在TCR活化后立即下降^[9]^。

CRBN通过影响活化T细胞的细胞核因子激活信号通路及表观遗传调控Kv1.3的表达来限制CD4^+^ T细胞的激活^[51]^。CRBN作为T细胞激活的重要拮抗剂，通过c端yippe-mis18基序与*Kcna*3基因（编码Kv1.3钾离子通道、T细胞钙内流，参与记忆T细胞介导的自身免疫性疾病的免疫调节）位点附近的3’保守区结合抑制T细胞的激活^[9, 51, 57]^。而在CRBN缺失的情况下，CD4^+^ T细胞在T细胞受体刺激下表现出活性增强及IL-2产生增加，最终导致钾通量和钙离子介导的信号转导增加，使得CD4^+^ T细胞过度活化。而过表达CRBN在T细胞中显著降低了Kv1.3 mRNA的表达，抑制T细胞的活性。研究^[51]^表明，T细胞特异性CRBN缺陷型小鼠的实验性自身免疫性脑脊髓炎可因Kv1.3引起的T细胞活化增加而加重。

### 2.2 CRBN调节T细胞的代谢与功能

CRBN缺失或CRBN E3连接酶调节化合物引发高代谢效应T细胞表型，Crbn^-^/^-^ CD8^+^ T细胞表现出超激活状态。抗CD3ε^+^抗CD28激活的Crbn^-^/^-^ CD8^+^ T细胞比活化的Crbn^+^/^+^CD8^+^ T细胞有更高的葡萄糖摄取、己糖激酶活性和NAD+/NADH比值，即增强了葡萄糖摄取率、基础糖酵解和呼吸速率。小鼠CD8^+^ T的Crbn细胞缺乏时，其中枢代谢表现为生物能量的升高，葡萄糖和氨基酸转运的超生理水平以及代谢酶的表达增加^[9, 51]^。

小鼠CRBN缺乏时，使得CD4^+^ T细胞分化为Th17效应细胞且IL-2产生增加^[9, 51]^。在Crbn^-^/^-^CD8^+^ T细胞与卵蛋白多肽TCR转基因小鼠（OT1）杂交的细胞对H-2Kb卵清蛋白四聚体免疫亲和力实验中发现，Crbn^-^/^-^CD8^+^ T细胞立即对高、中亲和力性多肽表达相当水平的CD69，但CD69在OT1表达时间更长。与低亲和力的多肽相比，高、中亲和力多肽促进OT1、Crbn^-^/^-^CD8^+^ T细胞的增殖。因此，CRBN控制抗原刺激后活化的T细胞的持续存在，但不会对低亲和力和无关抗原作出反应^[9]^。

## 3 CRBN调节剂治疗肺癌的研究进展

CRBN作为E3泛素连接酶复合物CRL4^CRBN^的底物受体，与来那度胺和波马那度胺等亚胺类免疫调节药物的靶点结合时，促进底物Ikaros和Aiolos招募到E3复合物中，从而导致底物泛素化和降解，进而调节T细胞的生长增殖，逆转PD-1抗体治疗肺癌的耐药^[58-61]^。而Ikaros和Aiolos是T细胞中IL-2表达的负调节因子^[62-64]^。如CRBN与CC-122结合，降解Aiolos和Ikaros，使得T细胞共刺激能力增强^[60]^。因此，调节CRBN的表达可增强效应T细胞功能，从而逆转耐药。多种CRBN调节剂现已用于临床，如一代药物沙利度胺和二代药物来那度胺等。沙利度胺等亚胺类药物可抑制TNF-*α*、IL-6和粒细胞-巨噬细胞集落刺激因子的生成，诱导IL-2介导的初级T细胞增殖，促进IFN的表达，刺激T淋巴细胞、细胞因子的产生和细胞毒性活性，从而增加T细胞的抗癌活性^[65]^。此外，不断有新型CRBN调节剂用于临床试验，如CC-885通过诱导CRBN和p97依赖的Polo样激酶1泛素化和降解，从而增强NSCLC对Volasertib的敏感性，与Volasertib协同抑制肺癌^[66, 67]^。

### 3.1 沙利度胺

有研究^[51, 58]^认为，当沙利度胺与CRBN结合时，抑制E3泛素连接酶复合物的形成，进而降低泛素连接酶的活性，导致四肢发育缺陷。最近的研究^[51]^表明，亚胺类免疫调节药物通过与CRBN结合时，改变了底物泛素化，刺激T细胞中IL-2的产生。沙利度胺与CRBN的C端结合，可以阻止CRBN与Kcna3（编码T细胞钙内流所需的Kv1.3钾离子通道）的结合，特异性地改变了*Kcna*3基因座的表观遗传修饰。长期沙利度胺的治疗增加了诱导CD4^+^ T细胞激活的Kv1.3的表达，减少了CRBN对Kcna3 R4的募集，从而增强T细胞的效应功能。

在2018年，有研究^[68]^将56例肺癌脑转移患者随机分为两组，每组28例。实验组在放疗基础上联合沙利度胺，观察两组的有效率及疾病进展时间。结果表明，实验组客观有效率为71.4%，对照组客观有效率为42.9%，差异有统计学意义（*P* < 0.05）。两组患者的中位疾病进展时间分别为8.2个月和5.9个月，差异有统计学意义（*P* < 0.05）。在2019年，将78例晚期NSCLC患者分为研究组（*n*=41）和对照组（*n*=37），研究组接受沙利度胺联合GP（吉西他滨+顺铂）方案化疗，对照组接受单纯GP方案化疗。随访18个月，观察并比较两组患者的卡氏体能状态（Karnofsky performance status, KPS）评分。治疗6个月和治疗12个月后，研究组患者的KPS评分均明显高于对照组（*P* < 0.05）^[69]^。两项研究均表明，沙利度胺可以有效地治疗肺癌。

### 3.2 来那度胺

免疫调节药物来那度胺最初被批准用于治疗骨髓增生异常综合征和多发性骨髓瘤^[70-72]^。研究^[73, 74]^表明，来那度胺具有抗血管生成和抗肿瘤活性并表现出免疫调节活性，调节细胞因子和生长因子的生成，如抑制TNF-*α*的生成并上调IL-10、IL-2和IFN-*γ*的生成。来那度胺还可以在T细胞受体激活后诱导T细胞增殖，是T细胞活化的有效共刺激因子，导致T细胞的细胞因子的增加和细胞毒性T细胞、CD8^+^ T细胞和NK细胞的活化^[72, 73, 75, 76]^。此外，来那度胺在没有免疫效应细胞的情况下也对肿瘤细胞有直接的抗增殖作用。在NSCLC中，已观察到以来那度胺为基础治疗的客观反应，提示来那度胺是治疗NSCLC的有效药物^[72]^。

将人NSCLC细胞系Lu-99、H1299、A549、EBC1和H460在37 oC含10%胎牛血清和抗生素的RMPI-1640培养液、5%CO_2_的加湿室中培养。用不同浓度的来那度胺接种于60 mm培养皿（2×10^5^个细胞）中孵育不同时间。在来那度胺处理后，使用TRIzol试剂从细胞中提取总RNA。结果显示，来那度胺可显著抑制NSCLC细胞（Lu-99、H1299、H460和A549）的增殖，且呈浓度依赖性^[72]^。

## 4 结语

CRBN作为E3泛素连接酶的底物，对其作用机制在多种肿瘤治疗与耐药中都进行了研究。CRBN可调节T细胞的生长、增殖和功能、调控肿瘤细胞的浸润和转移且延长无进展生存期。CRBN作为一种免疫调节药物分子靶点，具有多种潜在优势和发展潜力。关于CRBN的作用机制，CMs的调节作用和临床应用需要进一步研究和探讨，为充分利用CRBN功能调节T细胞和肿瘤细胞来提高免疫治疗药物疗效和逆转耐药提供更多可靠依据。
